# Une complication exceptionnelle de la pose d'une voie veineuse centrale jugulaire interne: pneumothorax, pneumo médiastin et retro pneumopéritoine et emphysème sous cutané géant

**DOI:** 10.11604/pamj.2015.20.226.4927

**Published:** 2015-03-12

**Authors:** Ahmed Belkouch, Rachid Sirbou, Saad Zidouh, Naoufal Chouaib, Mostafa Rafai, Lahcen Belyamani

**Affiliations:** 1 Service des Urgences Médico-Chirurgicales, Hôpital Militaire d'Instruction Mohammed V, Rabat, Maroc

**Keywords:** Pneumothorax, pneumo médiastin, retro pneumopéritoine, voie veineuse, Pneumothorax, pneumo mediastin, pneumoperitoneum, venous catheter

## Abstract

L'association: pneumothorax, pneumo médiastin, retro pneumopéritoine et emphysème sous cutané est connue de longue date comme complication de l'intubation et la ventilation mécanique, de l'endoscopie digestive ou de la chirurgie laparoscopique. En dehors de ce contexte, elle demeure inhabituelle surtout dans le cadre de La pose de voies veineuses centrales puisque le risque encouru est celui d'une brèche pleurale avec pneumothorax, il est surtout lié à la mise en place d'un cathéter central sous-clavier plus que lors de la pose d'une voie centrale par voie jugulaire. Nous rapportons le cas d'une patiente qui a souffert d'un pneumothorax associé à, un pneumo médiastin, un rétro pneumopéritoine et un emphysème sous cutané géant, suite à une tentative de catéthérisation de la veine jugulaire interne par voie postérieure. L'intérêt de cette observation réside dans la rareté exceptionnelle de cette association chez une patiente en ventilation spontanée et dans le mécanisme physiopathologique qu'elle suggère.

## Introduction

La pose d'une voie veineuse centrale est devenue une pratique courante au service des urgences. Sa maitrise par les urgentistes est donc une obligation. Cette maitrise compte aussi bien les connaissances pré-requises en anatomie, que la connaissance des différentes complications, leurs préventions et surtout leurs traitements.

## Patient et observation

Il s'agissait d'une patiente âgée de 42 ans, diabétique non insulinodépendante depuis 5 ans sous biguanide. Elle a été hospitalisée au service de médecine interne pour le bilan étiologique d'une altération de l’état générale avec des épisodes de fièvre inexpliquée. Le bilan avait conclu à une infection urinaire avec diabète décompensé. Un traitement antibiotique par voie par entérale a été initié. L'indication d'une voie centrale a été posée devant un état veineux périphérique médiocre. La patiente a été admise le lendemain matin à la salle de déchoquage du service des urgences pour dyspnée survenant après une tentative de prise d'une voie veineuse centrale jugulaire interne par voie postérieure. L'examen initial montrait une patiente avec une voie nasonnée, apyrétique et stable avec une tension artérielle à 110/66mmHg, fréquence cardiaque à 83 battement par minute, consciente et anxieuse, la fréquence respiratoire était à 24 cycle/ minute avec une saturation à 97% à l'air ambiant. L'examen clinique montrait un emphysème sous cutané généralisé et massif occupant la face, le cou, le thorax, l'abdomen et les racines des membres.

**Figure 1:**
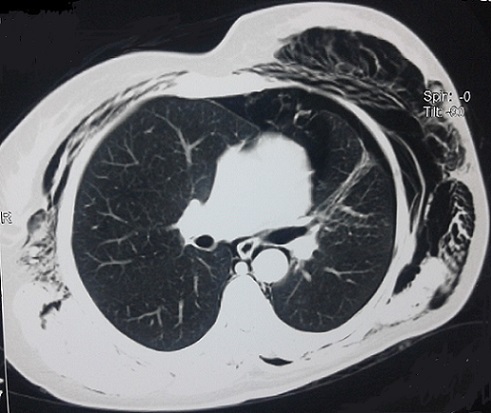
Coupe sous carinaire montrant le pneumothorax antérieur minime, le pneumo médiastin ainsi que l'emphysème sous cutané géant

**Figure 2:**
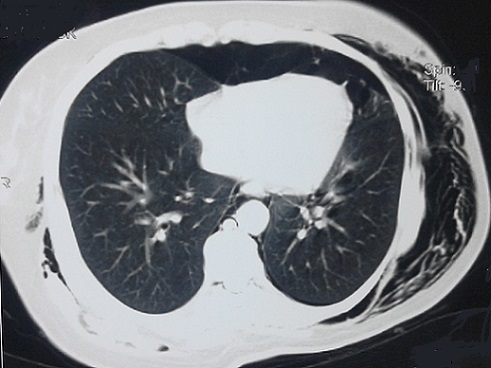
Pneumothorax antérieur minime et cloisonné

**Figure 3:**
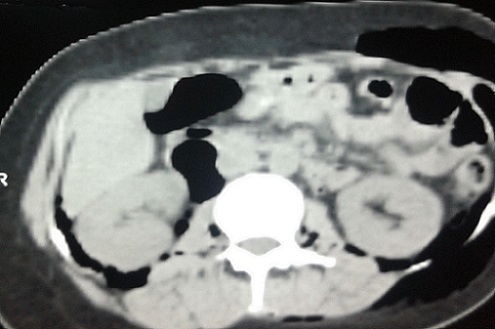
Diffusion de l’épanchement gazeux en rétro péritonéal et dans les lombes

La radio pulmonaire montrait un emphysème sous cutané diffus du cou et du tronc avec pneumothorax gauche de faible abondance. La tomodensitométrie montrait un emphysème sous cutané diffus des parties molles avec pneumothorax apicale gauche pneumo médiastin important (Figure 1 et Figure 2) ainsi qu'un retro pneumopéritoine étendu aux fosses iliaques (Figure 3). La recherche d'une autre étiologie pouvant expliquer ces épanchements gazeux est revenue négative notamment un examen ORL, fibroscopie digestive haute, et une bronchoscopie. L’évolution était favorable avec résolution de l'emphysème vers le 5éme jours après drainage thoracique.

## Discussion

Le mécanisme de diffusion des gaz au cours de la ventilation mécanique a été évoqué par Macklin en 1944, selon lui la pression téléxpiratoire positive induit une sur-distension puis la rupture des alvéoles provoquant une fuite des gaz dans l'intérstitium pulmonaire. La pression augmente à ce niveau et les gaz vont suivre les gaines péri bronchiques et péri vasculaires pour se diriger vers la plèvre et le médiastin. Le médiastin est un carrefour anatomique qui permet la diffusion vers la face par le biais des fascias cervicaux et vers le péritoine le long des gros vaisseaux qui traversent le diaphragme ou à travers les foramens de Morgagni ou de Bochdalek [1].

Au cours de la chirurgie laparoscopique, la diffusion du CO2 péritonéal peut se faire vers la plèvre et donner un pneumothorax soit du fait d'un traumatisme chirurgical du diaphragme et de la plèvre soit au travers d'un hiatus diaphragmatique congénital. Un Pneumopéritoine sous tension peut décoller l'espace tissulaire qui entoure l'oesophage et passer dans le médiastin. Lors des cures d'hernies inguinales, le gaz peut diffuser en rétropéritonéal puis le long des vaisseaux vers le médiastin. Le pneumomédiastin peut donner un pneumothorax par déchirure de la plèvre médiastinale [2]. Le CO2 suit les gaines du médiastin puis celles du cou avec possibilité d'infiltration du pharynx qui peut être à l'origine d'une obstruction des voies aériennes supérieures et des tissus sous-cutanés pour former un emphysème sous cutané [3].

L'endoscopie digestive peut se compliquer de perforations, Les facteurs incriminés dans la diffusion sont l'hyperpression exercée par l'endoscope ou par une insufflation excessive, si la perforation de l'oesophage se fait directement dans le thorax, celle post coloscopie peut aussi se produire directement dans l'espace retro péritonéal et diffuser à travers l'hiatus diaphragmatique le long des gros vaisseaux, responsable de l'apparition d'un pneumothorax, d'un pneumomédiastin, ou d'un emphysème sous-cutané [4]. Ce mécanisme de diffusion des gaz sous pression est également décrit dans de nombreuses observations de pneumomédiastin, pneumothorax et pneumopéritoine apparus au cours de l'utilisation d'une turbine fonctionnant avec de l'air et de l'eau pulsée pendant une extraction dentaire [5, 6].

Des cas sporadiques de diffusion étaient rapportés lors de l'administration d'o2 par sonde nasale en position sous muqueuse. L'absence de barrière anatomique réelle à la diffusion des gaz, explique le passage de l'oxygène des plans sous-muqueux rétro pharyngés vers le tissu sous-cutané (face, cou, partie antérieure du thorax) d'une part et le long de la trachée et de l'oesophage vers le médiastin antérieur d'autre part. À un stade ultérieur, lorsque la pression augmente, le gaz diffuse dans l'interstitium péri bronchique et vasculaire provoquant alors un décollement pleural; il diffuse également vers les cavités péritonéale et rétro péritonéale [7].

Le pneumothorax secondaire à La pose des voies veineuses centrales est dû à une brèche pleurale avec éruption d'air entre la plèvre viscérale et pariétale, ceci étant dû à la différence de pression entre le parenchyme pulmonaire et l'espace pleurale. Habituellement, l’évolution se fait: soit vers la fermeture de la brèche et l'arrêt d’échappement de l'air aboutissant à un décollement minime bien toléré cliniquement; soit au contraire, quand la fuite se pérennise ou son débit est fort, l'air va occuper tout l'espace pleurale avecà un stade ultime la survenue d'un collapsus pulmonaire et d'une tamponnade gazeuse. Dans la littérature 10% des pneumothorax s'accompagnaient d'un arrêt cardio circulatoire [8].

Dans la littérature Le risque de pneumothorax est évalué de 1 à 3%, il est surtout lié à la mise en place d'un cathéter central sous-clavier. Le risque de brèche pleurale est faible lors de la pose d'une voie centrale par voie jugulaire interne avec un taux inférieur à 0,5% [9].

Le taux de pneumothorax est théoriquement plus élevé par voie antérieur, à cause du trajet de l'aiguille, longitudinal et orienté vers le dôme pleural dans cette technique [10]. Au contraire, dans l'abord postérieur, le trajet de l'aiguille, bien plus transversal, met a priori à l'abri de tout pneumothorax [11].

Dans notre cas, la diffusion du pneumothorax s'est faite exceptionnellement vers le médiastin, et de là vers les régions cervico-faciales et retro péritonéale avec un emphysème sous cutané géant étendu de la face jusqu'aux racines des membres inférieurs. C'est une association rare, à notre connaissance, jamais décrite dans la littérature dans le cadre de la pose de voie jugulaire interne par voie postérieur et chez un malade en ventilation spontané, cette situation suggère fortement le fait que le pneumothorax se soit développé dans une plèvre cloisonnée canalisant ainsi l’épanchement en direction du hile pulmonaire puis vers le médiastin et de là vers le retro péritoine et les fascias du cou évitant l’évolution vers le pneumothorax compressif.

## Conclusion

La diffusion de l'air d'un pneumothorax iatrogène chez un malade non intubé et n'ayant pas subi une endoscopie ou une laparoscopie est une complication rare. Elle est habituelle en cas d'hyperpression exercée par le matériel utilisé au cours de la ventilation mécanique, de l'endoscopie ou de la laparoscopie. Cette observation décrit un cas exceptionnel de diffusion d'air qui a abouti à la constitution d'un pneumo médiastin, rétro pneumopéritoine et un emphysème sous cutané géant. L’évolution a été bénigne évitant la constitution un pneumothorax compressif. Elle s'explique probablement par l'existence d'une cloison pleurale qui a freinée cette diffusion vers la grande cavité pleurale.
